# Acantholytic squamous cell carcinoma of the lung with marked lymphogenous metastases and high titers of myeloperoxidase-antineutrophil cytoplasmic antibodies: a case report

**DOI:** 10.1186/s12885-018-4218-8

**Published:** 2018-03-16

**Authors:** Kenji Yorita, Kazuya Tsuji, Yoko Takano, Naoto Kuroda, Kei Sakamoto, Kaoru Arii, Yukio Yoshimoto, Kimiko Nakatani, Satoshi Ito

**Affiliations:** 1 Department of Diagnostic Pathology, Japanese Red Cross Kochi Hospital, 2-13-51, Shinhonmachi, Kochi-city, Kochi, 780-8562 Japan; 2 Department of Internal Medicine, Japanese Red Cross Kochi Hospital, 2-13-51, Shinhonmachi, Kochi-city, Kochi, 780-8562 Japan; 3 Department of Radiology, Japanese Red Cross Kochi Hospital, 2-13-51, Shinhonmachi, Kochi-city, Kochi, 780-8562 Japan

**Keywords:** Squamous cell carcinoma, Acantholytic squamous cell carcinoma, Lymphogenous metastasis, Small cell lung carcinoma, Myeloperoxidase-antineutrophil cytoplasmic antibody, Hypercalcemia, Parathyroid hormone–related protein

## Abstract

**Background:**

Acantholytic squamous cell carcinoma (ASQCC), histologically characterized by intercellular bridge loosening, is recognized as a rare variant of squamous cell carcinoma (SQCC). ASQCC may demonstrate a worse prognosis than conventional SQCC. Pulmonary ASQCC is particularly rare; its biological behavior and prognostic data have not been reported.

**Case presentation:**

We report the clinical and autopsy findings of a 71-year-old Japanese man with pulmonary ASQCC. Pulmonary lesions, suggestive of idiopathic interstitial pneumonia, were radiologically observed 3 and 6 years prior to the patient’s most recent hospitalization; however, the patient did not undergo further medical examinations. Upon being discovered unconscious, the patient was admitted to our hospital. Dehydration and lower limb muscle weakness were noted, as were laboratory findings of coagulation abnormalities and renal dysfunction. Computed tomography helped confirm a 21-mm peripheral nodule in the upper left lobe of the lung, with associated swollen lymph nodes in the bilateral hilar, mediastinal, and para-aortic regions. Brain and spinal lesions, suggestive of neurological disturbances, were not found. Small cell lung carcinoma was suspected, upon admission, but high serum levels of squamous cell carcinoma antigen and cytokeratin-19 fragments were present. Therefore, advanced lung cancer, possibly SQCC, was diagnosed. The patient was treated with best supportive therapy, and died one month after admission. Hypercalcemia and high serum levels of parathyroid hormone-related protein (PTHrP) and myeloperoxidase-antineutrophil cytoplasmic antibody (MPO-ANCA) titers were observed. Progressive renal insufficiency was absent due to improved renal function subsequent to hydration. An autopsy helped confirm the left lung tumor as an ASQCC associated with pulmonary lymphangitic carcinomatosis and multiple metastases in the lungs and lymph nodes. Skin lesions suggesting malignant tumors were absent. The metastatic lesions consisted largely of acantholytic tumor cells, and the lungs showed usual interstitial pneumonia pattern; vasculitis was absent.

**Conclusions:**

This is the first reported case of pulmonary ASQCC resulting in an aggressive clinical course, with marked lymphogenous metastases and PTHrP-associated hypercalcemia. The high serum MPO-ANCA titers were clinicopathologically insignificant, but may have been related to the pulmonary interstitial lesion. Pulmonary ASQCC represents a highly malignant subset of lung cancer.

## Background

Squamous cell carcinoma (SQCC) is characterized by stratified growth, but infrequently shows acantholysis (a loosening of the cell–cell contact). This results in adenoid (pseudoglandular, pseudoacinar) or pseudoangiosarcomatous growth patterns. SQCCs with acantholysis are rare, but the latest World Health Organization’s (WHO’s) skin [1], breast [2], oral cavity [3], and penis [4] blue books recognize such tumors as a histological subtype of SQCC. This is likely due to SQCC with acantholysis showing more aggressive behavior than conventional SQCC. However, whether cutaneous SQCCs forming adenoid patterns show worse prognoses than conventional SQCCs remains debatable.

The skin is the most frequent site of acantholytic tumors, with common skin pathology references [1, 5–8] classifying cutaneous SQCC with acantholysis as either acantholytic SQCC (ASQCC) and pseudovascular SQCC (PSQCC). The former has synonyms of adenoid or pseudoglandular SQCC, and the latter has synonyms of pseudoangiosarcomatous or pseudoangiomatous SQCC. ASQCC was first coined by Lever in 1947 [9], and comprises 2–4% of all cutaneous SQCCs [1]. Many common skin pathology textbooks histologically characterize ASQCC as adenoid (pseudoglandular) or pseudoacinar nests with central acantholysis and cohesive peripheral tumor cells [5–8]. The WHO classification of skin tumors [1] defines ASQCC as a loosening of intercellular bridges, with adenoid or pseudoacinar growth patterns being unnecessary. PSQCC is histologically characterized as SQCC with marked acantholysis, resulting in pseudovascular or pseudoangiosarcomatous growth. ASQCC and PSQCC are postulated to be overlapping entities [1] because both histological patterns share a common feature of tumor nests with central acantholysis and cohesive peripheral tumor cells. In the WHO classification of the breast [2], oral cavity [3], and penis [4], ASQCC or pseudoglandular carcinoma has been adopted as the histological name of SQCC with acantholysis, but this nomenclature appear to imply adenoid and pseudoangiosarcomatous growth of SQCC. PSQCC is not accepted as a separate entity in these organs, most likely due to its rarity. In this case report, we adopted the ASQCC and PSQCC definitions described in the WHO skin tumor classification [1].

Among primary lung cancer reports, 8 cases of SQCC with marked acantholytic changes have been reported, including 1 case of ASQCC [10, 11] and 7 cases of PSQCC [12–15]. Most pulmonary PSQCC cases showed poor prognoses, but the clinical course and biological behavior of pulmonary ASQCC has not been previously reported. Pulmonary PSQCC was included in the sarcomatoid carcinoma section of the previous WHO lung tumor classification [16], but neither PSQCC nor ASQCC were included in the latest (2015) WHO classification.

In this report, we describe the clinicopathologic and prognostic characteristics of pulmonary ASQCC found in our patient. We also discuss the aggressive nature of ASQCCs, the unique clinicopathologic features of our case, and our comprehensive literature review of the area.

## Case presentation

### Clinical and radiological findings

Six years prior to an emergency hospital admission, a 71-year-old Japanese man, with a 43-year history of smoking 2 packs/day, was diagnosed with bilateral pulmonary lesions. Radiographically, the lesions were suggestive of idiopathic interstitial pneumonia (IIP) because of the interstitial reticular markings at their peripheral and basal areas. Due to the patient’s stable condition, the lesions were only observed over time. Three years prior to admission, the 78-kg patient presented with a stomach ache, and declined a medical evaluation of the lung lesions. The patient did not undergo additional routine medical examination.

**Fig. 1:**
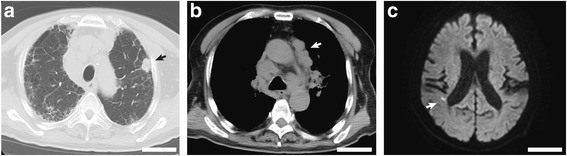
Radiologic images of the tumor and brain. **a**–**b** Axial computed tomography images show a 21-mm, solid, pulmonary nodule in the left upper lobe (**a**), arrow) and swollen mediastinal lymph nodes (**b**), arrow). **c** The axial diffusion-weighted magnetic resonance image shows a tiny high-intensity area (arrow) near the right-side ventricle in the cerebrum. Bars indicate 5 cm

Seven days before the emergency admission, the patient fainted in a public area, but did not seek medical help. Subsequently, the patient was discovered unconsciousness in his home, and was brought to our emergency department for evaluation. The patient had clouding of consciousness, and his weight had decreased to 58 kg. Marked oral dryness was observed, suggesting dehydration, with fine crackles heard upon auscultation. His reactions to light were normal and no facial nerve palsy was observed; left hemiparesis was suggested due to a positive pronator drift (Barré sign). Lower limb muscular weakness was also found. Upon admission, his laboratory results showed a mildly elevated white blood cell count, with abnormal renal function and coagulation indicated. Non-enhanced computed tomography (CT) imaging revealed a 21-mm pulmonary nodule in the left upper lobe (Fig. 1a), associated with enlarged lymph nodes in the mediastinum, bilateral pulmonary hilum, and para-aortic regions (Fig. 1b). The clinical diagnosis, upon admission, was advanced lung cancer, with small cell lung cancer (SCLC) being suspected. Diffusion-weighted magnetic resonance imaging showed tiny foci of cerebral infarctions, suggestive of Trousseau’s syndrome (Fig. 1c); however, no brain or spinal cord lesions causing the left hemiparesis and lower limb weakness were found. Further laboratory tests confirmed hypercalcemia (corrected calcium level, 11.1 mg/dL [normal, 8.5–10.5 mg/dL]), high serum levels of parathyroid hormone-related protein (PTHrP, 8.3 gmol/L [normal, < 1.1 pmol/L]), and normal Intact PTH levels. The serum levels of various tumor markers were elevated, including SQCC antigen, 39.7 ng/mL [normal, < 1.5 ng/mL]; cytokeratin-19 fragments, 393.6 ng/mL [normal, < 3.5 ng/mL]; and carcinoembryonic antigen (CEA), 10.7 ng/mL [normal, < 5 ng/mL]. To evaluate the cause of IIP, autoimmune disease was investigated using serological markers. Mildly elevated titers of antinuclear antibodies (1:80) and high titers of myeloperoxidase-antineutrophil cytoplasmic antibodies (MPO-ANCA, 71.4 enzyme-linked immunosorbent assay units (EU)/mL [normal, < 3.5 EU/ml]) were found, but proteinase 3-ANCA levels were within normal limits. Cytological specimens, prepared from bronchoalveolar lavage fluid, confirmed atypical squamoid cells. Therefore, advanced pulmonary SQCC associated with PTHrP-associated hypercalcemia and ANCA-associated vasculitis was clinically diagnosed. As contrast-enhanced CT and positron emission tomography were not performed to closely examine the distant metastasis, the tumor was classified as T1cN3cMx, more than Stage IIIB, according to the TNM classification of malignant tumors, eighth edition [17].

Best supportive care was chosen as the treatment course, after discussion with the patient’s son. Urine tests and creatinine levels improved after hydration, and the clinical features of rapidly progressive glomerulonephritis syndrome were absent. The patient’s serum corrected calcium level showed a gradual increase to a maximum of 20.2 mg/dL, two days prior to his death, which occurred one month after admission.

### Autopsy findings

**Fig. 2:**
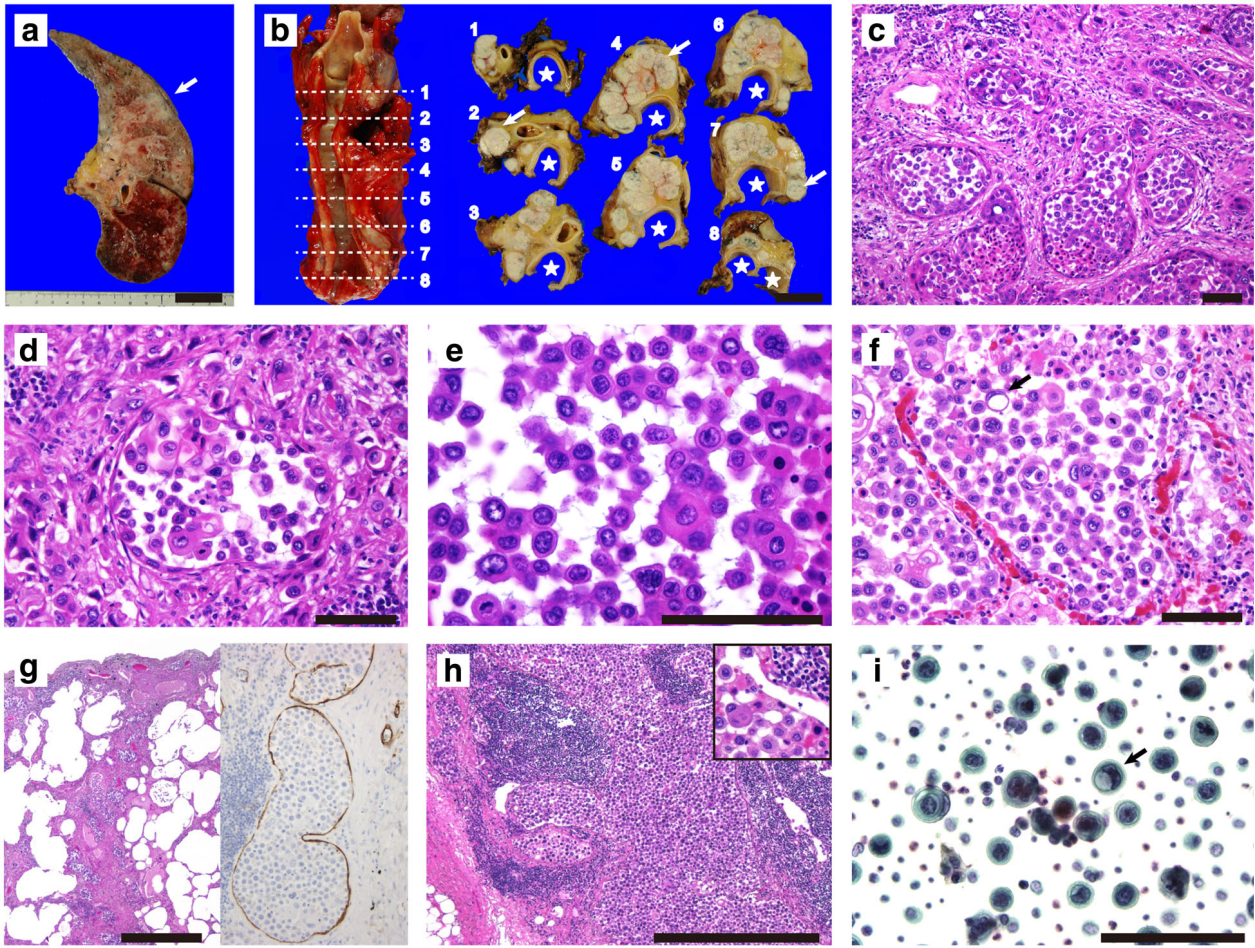
Autopsy and pathologic findings of the acantholytic squamous cell carcinoma of the lung. **a** An axially cut section of left lung shows the upper lobe tumor (an arrow) and hemorrhagic lower lobe. **b** Axially cut sections (numbers 1–8) of the mediastinum demonstrate metastatic lymph nodes (arrows) in the ipsilateral and contralateral sides. Stars indicate airway of the trachea and its bifurcation. The numbered, dotted lines correspond to the numbers of the cut sections. **c**–**d** The left upper lobe tumor includes pseudoacinar nests containing discohesive atypical cells. Individual atypical tumor cells are also seen in the stroma (**d**). **e**–**i** The monomorphic and discohesive tumor cells that are associated with squamoid cells or nests (**e**) show filling in some alveolar spaces (**f**), pulmonary lymphangitis carcinomatosis (**g**), left, low magnification; right, D2–40 immunostained section), and lymph node metastasis (**h**) and its inset, high magnification of (**h**). The papanicolaou-stained smears (**I**) prepared from bilateral pulmonary effusion confirm acantholytic tumor cells including signet-ring cells (arrow) that are also seen in (**f**) (arrow). Images of (**c**–**h**) are taken from sections stained with hematoxylin and eosin. Bars indicate 3 cm in (**a**–**b**), 100 μm in (**c**–**f**), and (**i**), and 1 mm in (**g**) and (**h**)

Approximately 3 h after the patient’s death, an autopsy was performed after securing his son’s permission. Per the son’s request, the central nervous system was not examined. Skin lesions suggestive of malignant tumors were absent. A 23-mm left upper lung lobe tumor was confirmed with sections cut (Fig. 2a), and multiple, vague, nodular lesions that suggested metastasis were present. Honeycombing was seen in the inferior and posterior peripheral regions of the lungs. Contralateral and ipsilateral mediastinal lymph nodes (Fig. 2b) and bilateral hilar lymph nodes were markedly enlarged; swollen lymph nodes were also noted around the aorta. Cardiac effusion was not calculated due to the presence of adhesive pericarditis. No abnormal findings were observed in other organs.

### Pathological findings

Histologically, the left upper lobe tumor included adenoid or pseudoacinar nests containing discohesive, round, or polygonal atypical cells (Fig. 2c–d); pseudovascular arrangements of the tumor cells were not observed. Individual tumor cells, varying in size and shape, invaded the fibrous stroma (Fig. 2d). A diffuse appearance of the discohesive, monotonous cells, without adenoid or pseudoacinar pattern, was noted (Fig. 2e), and a large portion of them filled the alveoli, suggesting that they spread through air spaces (Fig. 2f). They also demonstrated prominent lymphatic permeation, forming pulmonary lymphangitis carcinomatosis (PLC; Fig. 2g). The discohesive monotonous cells were associated with atypical squamoid cells (Fig. 2d–f) and small numbers of signet-ring atypical cells (Fig. 2f). Extrapleural extension of the tumor cells was not observed. The clear cytoplasm of the signet-ring tumor cells was negative for periodic acid-Schiff (PAS), diastase-PAS, Alcian blue, and adipophilin (clone AP125).

**Fig. 3:**
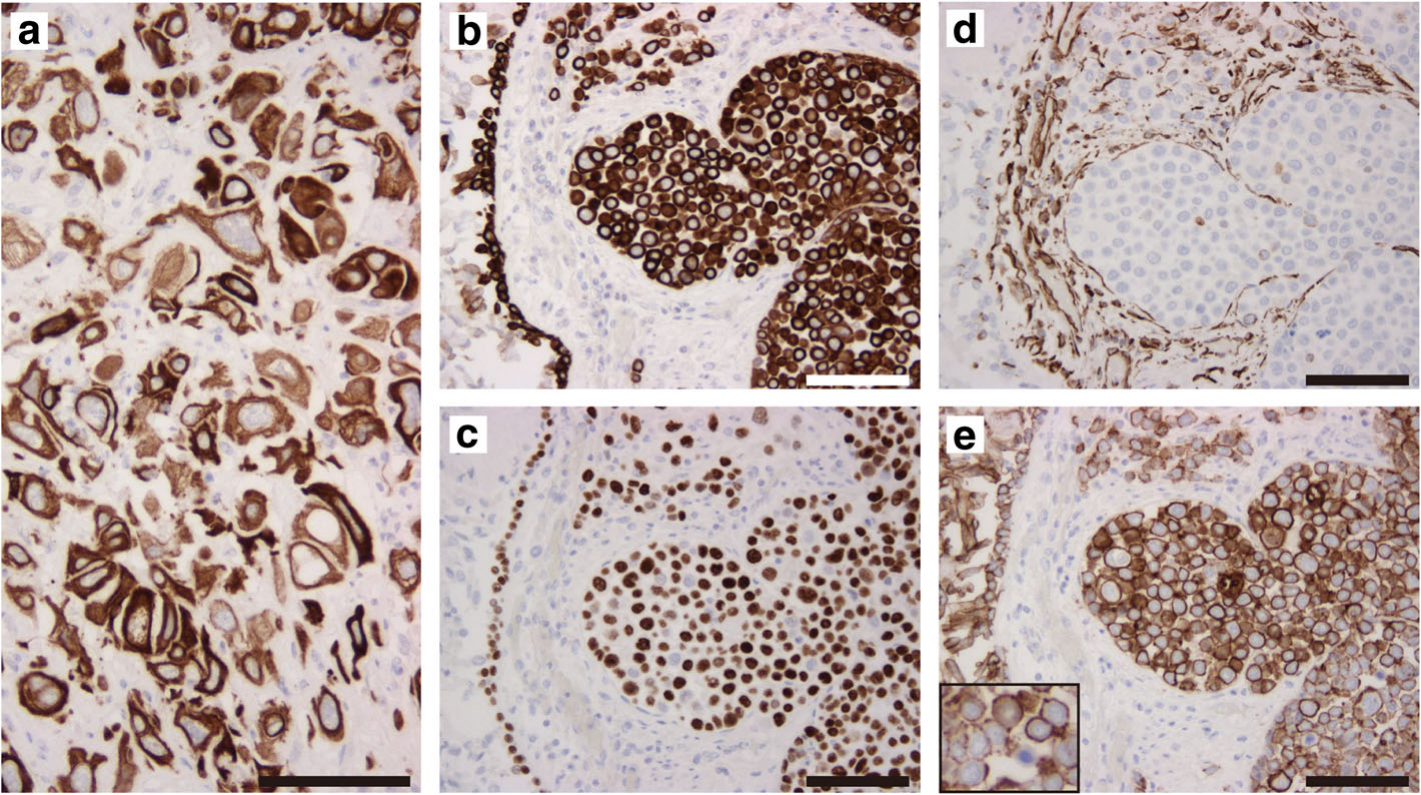
Immunohistochemistry of the acantholytic squamous cell carcinoma of the lung. **a** The pleomorphic tumor cells are diffusely positive for cytokeratin 5 (CK5). **b**–**e** The monomorphic, acantholytic tumor cells, mainly seen in lymphatic duct of the bronchial wall, are diffusely positive for CK5 (**b**) and p40 (**c**) and almost negative for vimentin (**d**). The acantholytic tumor cells are largely positive for E-cadherin (**e**), but the low intensity of the cellular membrane is notable (inset, high magnification of (**e**). The bronchial epithelium (left side of (**b**–**e**) is an internal positive control for CK5 (**b**), p40 (**c**), and E-cadherin (**e**) and the bronchial stromal cells are internal positive controls for vimentin. Bars indicate 100 μm

Immunohistochemically, the tumor cells, described above, were diffusely positive for cytokeratin 5 (clone XM26, Fig. 3a and b) and p40 (clone BC28, Fig. 3c), and were negative for cytokeratin 7 (clone OV-TL 12/30), thyroid transcription factor 1 (clone 8G7G3/1), Napsin A (polyclonal), desmin (clone D33), alpha-smooth muscle actin (clone 1A4), anaplastic lymphoma kinase (ALK, clone 5A4), and nuclear protein in testis (NUT, clone C52B1). The monomorphic discohesive tumor cells and squamoid foci were mostly negative for vimentin (clone V9, Fig. 3d), focally positive for CEA (clone II7), and largely positive for E-cadherin (clone NCH-38, Fig. 3e). In total, the left upper lobe tumor showed a mixture of ASQCC and poorly differentiated SQCC, with both components appearing equally prevalent.

Multiple vague, intrapulmonary masses and lymph node metastases (Fig. 2h) in the bilateral hilar, mediastinal, para-aortic regions, and non-regional areas consisted of diffuse acantholytic monomorphic tumor cells with foci of well to poorly differentiated SQCC. Adenoid or pseudoacinar nests were rather minor. Neoplastic pericarditis, with ASQCC cells, induced adhesion of the visceral and parietal pericardial layers. Cytology specimens prepared from the bilateral pulmonary effusion also confirmed the presence of acantholytic tumor cells (Fig. 2i). Blood-borne metastasis was inconspicuous, but some acantholytic tumor cells were seen in the renal corpuscles. The acantholytic component of the tumor apparently worsened the patient’s prognosis.

ANCA-related vasculitis, such as necrotizing vasculitis, was absent in the lungs, kidneys, and other organs. The bilateral lungs showed the usual interstitial pneumonia (UIP) pattern, edema, and small foci of acute pneumonia. The bilateral kidneys showed proximal renal tubule degeneration, compatible with prerenal failure induced by dehydration, probably due to hypercalcemia. Crescent formation in the glomeruli was present in < 0.5% of these structures. The heart and its valves showed neither thrombotic endocarditis nor vegetations. Malignant tumors, except for the lung cancer, were absent.

## Discussion and conclusions

The present patient demonstrated pulmonary ASQCC that resulted in an aggressive clinical course with marked lymph node metastases and PLC. Although neither contrast-enhanced CT or positron emission tomography were performed, we believe that the present tumor was a primary lung cancer because CT and the autopsy evaluated most of the primary SQCC sites, including the head, neck, skin, and esophagus.

The histological diagnosis of SQCC was not difficult because of the diffuse immunoreactivity to cytokeratin 5 and p40, although sarcomatoid carcinoma was confirmed via a differential diagnosis. NUT carcinoma could have been included in the differential diagnoses as the tumor showed squamous differentiation and possible discohesive growth [18], but our case was immunohistochemically negative for NUT. The clinical diagnosis of the malignant tumor was straight-forward, showing marked lymph node metastases and high serum values for SQCC markers. A mildly elevated serum CEA level was also observed, but this phenomenon was expected due to the acantholytic component showing focal CEA immunoreactivity; ASQCC cell lines expressing CEA have been reported [19]. Other clinically unique features included the tiny cerebral lesions, PTHrP-associated hypercalcemia, high MPO-ANCA titers, and marked lymphogenous metastasis.

Among 3426 autopsy cases involving malignant tumors, cerebral infarctions were observed in 256 cases (7%), with the most common etiology being non-bacterial thrombotic endocarditis [20], which was not observed in our case. In ischemic stroke patients with underlying malignancies, lung cancer is the most frequent tumor type [21, 22]. Therefore, our case may be indicative of cancer-related hypercoagulability, or Trousseau’s syndrome. However, Trousseau’s syndrome appeared unlikely because the radiological features of the patient’s tiny cerebral infarctions were not typical of embolic stroke, and the malignancy hypercoagulable state typically occurs in mucin-producing adenocarcinomas [23]; hypercoagulability associated with SQCC has rarely been reported. The patient’s hypercalcemia and dehydration were likely hypercoagulation related because hypercalcemia can accelerate clot formation [24] and dehydration induces hyper-viscosity.

Takai et al. reported hypercalcemia is 17 of 690 cases (2.4%) patients with lung carcinoma, and SQCC is the most frequent histology of lung cancer associated with hypercalcemia [25]. As in the present case, lung cancers with hypercalcemia are mostly related to elevated PTHrP [26], and treatment of the lung cancer can normalize both serum calcium and PTHrP levels [25]. An ASQCC literature review showed only one case accompanied by hypercalcemia in the end stage, but the serum PTHrP value was not evaluated [27]. With respect to MPO-ANCA, 8 cases of lung carcinoma (SQCC, 2 cases; adenocarcinoma, 6 cases) with high titers of MPO-ANCA have been reported [28, 29]. Six of these cases showed renal impairment, and 4 (SQCC, 2 cases; adenocarcinoma, 2 case) demonstrated normalized MPO-ANCA levels, after cancer treatment. ASQCCs with high titers of MPO-ANCA have not been previously reported. In our case, the MPO-ANCA level was clinicopathologically insignificant, regardless of the high titers. However, Arulkumaran et al. reported that UIP is the most common histological pattern in interstitial lung disease with MPO-ANCA-associated vasculitis [30]. Ando et al. also reported that 6 of 58 patients with IIP showed MPO-ANCA-positive conversion within 11–71 months (mean, 45 months) of the IIP diagnosis, and 4 of those patients did not develop microscopic polyangiitis during the follow-up [31]. Our case may be similar to those 4 cases [31].

The present case had N3 lymph node metastases, although the primary lung tumor was T1c, which resulted in a radiological diagnosis of SCLC. This is rare because among 5415 cases of T1c lung cancers, only 13 (0.24%) demonstrated N3 lymph node metastasis [32]. In a previous report, primary lung SQCC with PLC was infrequent [33]. In that report, 29 of 34 cases (85%) of primary lung cancer with PLC were diagnosed with adenocarcinoma and 2 cases (6%) were diagnosed with SQCC. To our knowledge, T1 SQCC with N3 lymph node metastasis and PLC has not been previously reported.

From our literature review, 9 cases of pulmonary SQCC with discohesive tumor nests, including our case, have been reported (Table 1) [10–15]. The mean patient age was 63 (range, 47–79) years and the male to female ratio was 8 to 1. Seven cases showed pseudovascular or pseudoangiosarcomatous features [12–15], corresponding to PSQCC of the skin [1]. In PSQCC of the lung, metastasis was present in 5 of 7 cases, and blood-borne metastasis was noted rather than lymphogenous metastasis. This is due to the sites of metastasis including the opposite lung, liver, bones, and adrenal glands, but not the lymph nodes [13, 15]. The prognosis of pulmonary PSQCC appears to be poor since 5 of the 7 patients died within 3 years of their diagnosis [13, 15]; the survival of the remaining 2 patients was unreliable due to a follow-up period of only 1 year [12, 14]. In contrast, only 1 case of pulmonary ASQCC has been reported [10, 11]. As was seen in our case, the first case of ASQCC showed diffuse fashion of acantholytic tumor cells. Adenoid or pseudoacinar tumor growth was not described in the first case and was minor in our case. Therefore, adenoid or pseudoacinar growth may be unnecessary for defining pulmonary ASQCC. The first pulmonary ASQCC case did not describe lymphovascular invasion, metastasis, or the prognosis. Our case showed an aggressive phenotype, with marked lymphogenous metastases. The multiple intrapulmonary metastases might represent aerogenous metastases with the primary lesion’s acantholytic tumor cells spreading through the air spaces. ASQCC and PSQCC of the lung might demonstrate different mechanisms of metastasis and may require different clinical interventions. Further studies of pulmonary ASQCC will be required to clarify its biological behavior.

**Table 1:** Literature review of cases of pulmonary squamous cell carcinoma with acantholysis

First author (Year published)	Age/ gender	Smoking	Tumor site	Maximum size (mm)	Histology		ly/v	Metastasis	Stage at the initial diagnosis	Diagnostic modality and treatment	Prognosis(months)
					Acantholytic component (%)	Other tumor component (%)					
Banerjee (1992) [12]	63/M	NA	LL	NA	Pseudoangio–sarcomatous or pseudovascular(%, NA)	Solid sheets of cohesive, large, undifferentiated pleomorphic cells (%, NA)	NA/NA	No metastasis	NA	Biopsy, RT	Alive (1-year follow–up)
Nappi (1994) [13]	47/M	Long–term cigarette smokers	RU	50		Small areas of SQCC (%, NA)	(−)/(−)	The opposite lung, liver, bones, and adrenal glands	Stage I	Lobectomy and RT	DOD (20 mo)
	48/M			45							DOD (34 mo)
	54/F		RU + RM	70					Stage III	Wedge biopsy and RT	DOD (5 mo)
Smith (1999) [14]	66/M	NA	RU	70		NA	NA/NA	NA	NA	Core needle biopsy, neoadjuvant CRT, and surgery (no viable tumor cells)	Alive (a few mo after surgery)
Kong (2011) [15]	79/M	1/2 pack per day for 60 years	LU	50		NA		Adrenal glands (1 mo after diagnosis)		Biopsy, supportive treatment	DOD (2 mo)
	76/M	1/2 pack per day for 20 years	RU	60		Small nests of SQCC (%, NA)		Ribs, no LN metastasis		Lobectomy, lymphadenectomy, and CT	DOD (3 mo)
Park (2016) and Choi (2016) [11]	64/M,	35 pack–year ex–smoker	LU	29	Acantholytic (> 99%)	Sheets of SQCC (< 1%)		NA		Lobectomy with mediastinal LN dissection	NA
Present case	71/M	2 packs per day for 43 years		23	Acantholytic (50%, the primary site; 60%, metastatic sites)	SQCC, por (50%, the primary site; 20%, metastatic sites), SQCC, well to mod (20%, metastatic sites)	(+++)/(+)	LN metastases (bilateral hilar, mediastinal, paraaortic regions)	More than Stage IIIB	None (best supportive care)	DOD (1 mo)

*Abbreviations*: *M*, male; *F*, female; *NA*, not available; *LL*, left lower lobe; *RU*, right upper lobe; *RM*, right middle lobe; *LU*, left upper lobe; *SQCC*, squamous cell carcinoma; por, poorly differentiated; *well*, well differentiated; *mod*, moderately differentiated; *ly*/v, lymphatic and vascular invasion; (−), absent; (+), present; (+++), markedly present; *mo*, month or months; *LN*, lymph node; *RT*, radiotherapy; *CRT*, chemoradiotherapy; *CT*, chemotherapy; *DOD*, died of disease

Although ASQCC in our case showed aggressive behavior, the prognosis of cutaneous ASQCC is open to debate [34–38]. That review of prognostic studies of cutaneous ASQCCs examined tumor histologies showing adenoid or pseudoacinar patterns. As observed in the present report, the 4 cases of SQCC showed diffuse growth of acantholytic tumor cells, with the prognostic data also reported [27, 39, 40]. Most of the cases showed aggressive clinical courses (3 skin and 1 esophageal cases); 3 of the 4 cases showed lymph node metastasis, and all 3 cutaneous cases died of the disease within 8 months of diagnosis, despite surgery and/or radiotherapy being performed. Therefore, the diffuse presentation of acantholytic SQCC cells, without forming adenoid or pseudoacinar patterns, may influence the cancer’s aggressive biological behavior.

Five published cases, similar to our case, demonstrated pathology that included acantholytic cells with signet-ring morphology [10, 11, 27, 39, 40]. Our case demonstrated acantholytic signet-ring tumor cells in either the alveolar spaces or the pulmonary effusion. A literature review showed that SQCC with signet-ring morphology in the lung [10, 11, 41], pyriform sinus [42], esophagus [40], and skin [27, 39, 43] often presented as ASQCC. The signet-ring morphology suggests primary and metastatic adenocarcinoma because they are seen in ALK-mutated lung cancers and adenocarcinomas of various sites, especially the stomach. In our case, the tumor cells did not exhibit mucin or immunohistochemical expression of pulmonary adenocarcinoma markers and ALK.

Considering that acantholytic tumor cells can be seen along the invasive front of SQCC [40, 44], the poorly differentiated SQCC component of the primary tumor may be a source of the acantholytic tumor cells in the present case. In addition, our case showed metastatic lymph nodes containing acantholytic tumor cells with foci of well to poorly differentiated SQCC, with or without keratin pearls, despite the primary tumor showing a paucity of keratin formation. This fact suggests that acantholytic tumor cells indicate a transition to SQCC at the metastatic sites. We postulate that SQCC cells can transition into ASQCC, and vice versa, according to the cells’ microenvironments. ASQCC is probably induced by the decreased presence of adhesion molecules, and we immunohistochemically showed decreased cell membrane expression of E-cadherin on acantholytic tumor cells. Griffin et al. reported that the loss of E-cadherin and desmoglein 3 expression is seen in cutaneous ASQCCs [45]. In our case, the ASQCC cells might have adapted to, and proliferated in, the fluid environment because they were mainly localized in the lymphatic ducts and lymph node sinuses, as well as in the pleural effusion and alveolar spaces with pulmonary edema. Discohesive carcinoma cells may have increased motility and mesenchymal properties, as often seen in carcinomas showing epithelial mesenchymal transitions. Acantholytic tumor cells of SQCCs that are immunoreactive to vimentin have been reported in the skin (*n* = 2) [39], penis (*n* = 1) [46], and aerodigestive tract (*n* = 4) [47]. However, the acantholytic tumor cells in our patient did not show either apparent spindle cells or rhabdoid morphology, and were mostly negative for vimentin and smooth muscle markers. Pyriform sinus ASQCCs, histologically similar to our case, have also shown to be negative for vimentin and desmin [42]. Therefore, whether ASQCCs must consistently show mesenchymal properties is debatable.

In this case report, we described a novel case of pulmonary ASQCC that showed an aggressive clinical course, PLC, prominent lymphogenous metastases, and hypercalcemia related to high serum levels of PTHrP. To our knowledge, the present report describes the second case of pulmonary ASQCC, but is the first to show the tumor’s biological behavior and prognosis. Of note, the bulky lymph node metastases observed in the hilar and mediastinal regions radiologically mimicked SCLC. Acantholytic tumor cells, with characteristic diffuse morphology, were the main histological feature of the primary and metastatic sites. Vasculitis was absent, despite the high MPO-ANCA titers. Further studies are needed to clarify the clinicopathological characteristics of this rare malignant tumor.

## Abbreviations

ALK: Anaplastic lymphoma kinase
ASQCC: Acantholytic squamous cell carcinoma
CEA: Carcinoembryonic antigen
CT: Computed tomography
IIP: Idiopathic interstitial pneumonia
MPO-ANCA: Myeloperoxidase-antineutrophil cytoplasmic antibody
NUT: Nuclear protein in testis
PAS: Periodic acid-Schiff
PLC: Pulmonary lymphangitis carcinomatosis
PSQCC: Pseudovascular SQCC
PTHrP: Parathyroid hormone related protein
SCLC: Small cell lung carcinoma
SQCC: Squamous cell carcinoma
UIP: Usual interstitial pneumonia
WHO: World Health Organization

## References

[1] Weedon D, Morgan MB, Gross C, Nagore E, Yu LL, LeBoit PE, Burg G, Weedon D, Sarasin A (2005). Squamous cell carcinoma. World Health Organization classification of skin tumors.

[2] Reis-Filho JS, Lakhani SR, Gobbi H, Sneige N, Lakhani SR, Ellis LO, Schnitt SJ, Tan PH, van de Vijver MJ (2012). Metaplastic carcinoma. World Health Organization classification of the breast.

[3] Sloan P, Nylander K, Gale N, Reibel J, Hunter K, Salo T, El-Naggar AK, Chan JKC, Grandis JR, Takata T, Slootweg PJ (2017). Malignant surface epithelial tumors. World Health Organization classification of head and neck tumors.

[4] Cubilla AL, Dillner J, Amin MB, Moch H, Ayala A, Sanchez DF, Moch H, Humphrey PA, Ulbright TM, Reuter VE (2016). Malignant epithelial tumors. World Health Organization classification of tumors of the urinary system and male genital organs.

[5] Kirkham N, Aljefri K, Elenitsas R, Rosenbach M, Murphy GF, Rubin AI, Xu X (2015). Tumors and cysts of the epidermis. Lever’s histopathology of the skin.

[6] Patterson J, Wick M (2006). Nonmelanocytic tumors of the skin. Atlas of tumor pathology.

[7] Luzar B, Calonje E, Bastian B. Tumors of the surface epithelium, In: Calonje E, Brenn T, Lazar A, Mckee PH. McKee’s pathology of the skin. 4 , 2. China: Elsevier-Saunders; 2012. p. 1076–1149.

[8] Patterson JW, Patterson JW (2016). Tumors of the epidermis. Weedon’s skin pathology.

[9] Lever WF (1947). Adenocanthoma of sweat glands; carcinoma of sweat glands with glandular and epidermal elements: report of four cases. Arch Dermatol Syphilol.

[10] Park HS, Lee S (2016). Acantholytic squamous cell carcinoma of the lung showing significant signet ring cell component. Histopathology.

[11] Choi SE, Park HS (2016). Pulmonary acantholytic squamous cell carcinoma with focal signet ring cell morphology mimicking malignant mesothelioma on fine needle aspiration cytology: a case report. Cytopathology.

[12] Banerjee SS, Eyden BP, Wells S, McWilliam LJ, Harris M (1992). Pseudoangiosarcomatous carcinoma: a clinicopathological study of seven cases. Histopathology.

[13] Nappi O, Swanson PE, Wick MR (1994). Pseudovascular adenoid squamous cell carcinoma of the lung: clinicopathologic study of three cases and comparison with true pleuropulmonary angiosarcoma. Hum Pathol.

[14] Smith AR, Raab SS, Landreneau RJ, Silverman JF (1999). Fine-needle aspiration cytologic features of pseudovascular adenoid squamous-cell carcinoma of the lung. Diagn Cytopathol.

[15] Kong M, Ren X, You Q, Yao H, Teng X (2011). Pseudoangiosarcomatous squamous cell carcinoma of the lung. J Int Med Res.

[16] Corrin B, Wick MR, Chang YL, Nappi O, Rossi G, Finkelstein SD, Travis WD, Brambilla E, Muller-Hermelink HK, Harris CC (2004). Sarcomatoid carcinoma. World Health Organization classification of lung, pleura, thymus and heart.

[17] Lung I, Brierley JD, Gospodarowicz MK, Wittekind C (2017). TNM classification of malignant tumors.

[18] Policarpio-Nicolas ML, de Leon EM, Jagirdar J (2015). Cytologic findings of NUT midline carcinoma in the hilum of the lung. Diagn Cytopathol.

[19] Yanagawa T, Hayashi Y, Yoshida H, Yura Y, Nagamine S, Bando T (1986). An adenoid squamous carcinoma-forming cell line established from an oral keratinizing squamous cell carcinoma expressing carcinoembryonic antigen. Am J Pathol.

[20] Graus F, Rogers LR, Posner JB (1985). Cerebrovascular complications in patients with cancer. Medicine (Baltimore).

[21] Taccone FS, Jeangette SM, Blecic SA (2008). First-ever stroke as initial presentation of systemic cancer. J Stroke Cerebrovasc Dis.

[22] Cestari DM, Weine DM, Panageas KS, Segal AZ, DeAngelis LM (2004). Stroke in patients with cancer: incidence and etiology. Neurology.

[23] Varki A (2007). Trousseau's syndrome: multiple definitions and multiple mechanisms. Blood.

[24] Hilgard P (1973). Experimental hypercalcaemia and whole blood clotting. J Clin Pathol.

[25] Takai E, Yano T, Iguchi H, Fukuyama Y, Yokoyama H, Asoh H (1996). Tumor-induced hypercalcemia and parathyroid hormone-related protein in lung carcinoma. Cancer.

[26] Kanaji N, Watanabe N, Kita N, Bandoh S, Tadokoro A, Ishii T (2014). Paraneoplastic syndromes associated with lung cancer. World J Clin Oncol.

[27] Cramer SF, Heggeness LM (1989). Signet-ring squamous cell carcinoma. Am J Clin Pathol.

[28] Chemouny JM, Pagnoux C, Caudwell V, Karras A, Borie R, Guillevin L (2014). ANCA-associated diseases and lung carcinomas: a five-case series. Clin Nephrol.

[29] Okauchi S, Tamura T, Kagohashi K, Kawaguchi M, Satoh H (2016). Elevated serum levels of two anti-neutrophil cytoplasmic antibodies in a lung cancer patient: a case report. Biomed Rep.

[30] Arulkumaran N, Periselneris N, Gaskin G, Strickland N, Ind PW, Pusey CD (2011). Interstitial lung disease and ANCA-associated vasculitis: a retrospective observational cohort study. Rheumatology (Oxford).

[31] Ando M, Miyazaki E, Ishii T, Mukai Y, Yamasue M, Fujisaki H (2013). Incidence of myeloperoxidase anti-neutrophil cytoplasmic antibody positivity and microscopic polyangitis in the course of idiopathic pulmonary fibrosis. Respir Med.

[32] Goldstraw P, Chansky K, Crowley J, Rami-Porta R, Asamura H, Eberhardt WE (2016). The IASLC lung Cancer staging project: proposals for revision of the TNM stage groupings in the forthcoming (eighth) edition of the TNM classification for lung Cancer. J Thoracic Oncol.

[33] Yang SP, Lin CC (1972). Lymphangitic carcinomatosis of the lungs. The clinical significance of its roentgenologic classification. Chest.

[34] Stratigos A, Garbe C, Lebbe C, Malvehy J, del Marmol V, Pehamberger H (2015). Diagnosis and treatment of invasive squamous cell carcinoma of the skin: European consensus-based interdisciplinary guideline. Eur J Cancer.

[35] Nappi O, Pettinato G, Wick MR (1989). Adenoid (acantholytic) squamous cell carcinoma of the skin. J Cutan Pathol.

[36] Pyne JH, Myint E, Barr EM, Clark SP, David M, Na R (2017). Acantholytic invasive squamous cell carcinoma: tumor diameter, invasion depth, grade of differentiation, surgical margins, perineural invasion, recurrence and death rate. J Cutan Pathol.

[37] Ogawa T, Kiuru M, Konia TH, Fung MA (2017). Acantholytic squamous cell carcinoma is usually associated with hair follicles, not acantholytic actinic keratosis, and is not’high risk’: diagnosis, management, and clinical outcomes in a series of 115 cases. J Am Acad Dermatol.

[38] Garcia C, Crowson AN (2011). Acantholytic squamous cell carcinoma: is it really a more-aggressive tumor?. Dermatol Surg.

[39] Ikegawa S, Saida T, Takizawa Y, Tokuda Y, Ito T, Fujioka F (1989). Vimentin-positive squamous cell carcinoma arising in a burn scar. A highly malignant neoplasm composed of acantholytic round keratinocytes. Arch Dermatol.

[40] Lee D (2016). Acantholytic squamous cell carcinoma of the oesophagus with prevalent single isolated tumour cells including signet ring cells and many osteoclast-like giant cells. Pathology.

[41] Yigit N, Celik E, Yavan I. Prominent signet ring cell morphology in a pulmonary squamous cell carcinoma. Turk Patoloji Derg. 2017; 10.5146/tjpath.2015.01337. 10.5146/tjpath.2015.01337 28272660

[42] Klijanienko J, Micheau C, Ponzio-Prion A, Azli N (1990). Signet-ring squamous cell carcinoma of the pyriform sinus. Am J Clin Pathol.

[43] McKinley E, Valles R, Bang R, Bocklage T (1998). Signet-ring squamous cell carcinoma: a case report. J Cutan Pathol.

[44] Watanabe K, Mukawa A, Saito K, Nakanishi I, Tsuda H (1986). Adenoid squamous cell carcinoma of the skin overlying the right breast. An autopsy case clinically manifested with rapid growth and widely spreading metastases. Acta Pathol Jpn.

[45] Griffin JR, Wriston CC, Peters MS, Lehman JS (2013). Decreased expression of intercellular adhesion molecules in acantholytic squamous cell carcinoma compared with invasive well-differentiated squamous cell carcinoma of the skin. Am J Clin Pathol.

[46] Chavan RY, Bali A, Savita KS, Chethan JV (2014). Vimentin positive acantholytic penile squamous cell carcinoma with rhabdoid features. J Cancer Res Ther.

[47] Gu X, Jiang R, Fowler MR (2012). Acantholytic squamous cell carcinoma in upper aerodigestive tract: histopathology, immunohistochemical profile and epithelial mesenchymal transition phenotype change. Head Neck Pathol.

